# An online international comparison of palliative care identification in primary care using the Surprise Question

**DOI:** 10.1177/02692163211048340

**Published:** 2021-10-01

**Authors:** Nicola White, Linda JM Oostendorp, Victoria Vickerstaff, Christina Gerlach, Yvonne Engels, Maud Maessen, Christopher Tomlinson, Johan Wens, Bert Leysen, Guido Biasco, Sofia Zambrano, Steffen Eychmüller, Christina Avgerinou, Rabih Chattat, Giovanni Ottoboni, Carel Veldhoven, Patrick Stone

**Affiliations:** 1 Marie Curie Palliative Care Research Department, Division of Psychiatry, University College London, London, UK; 2 Primary Care and Population Health, Institute of Epidemiology and Health Care, University College London, London, UK; 3 Palliative Care Unit, Department of Oncology, Hematology and BMT, and Pneumology, University Medical Center Hamburg-Eppendorf, Hamburg, Germany; 4 Interdisciplinary Palliative Care Unit, Department of Hematology, Oncology, and Pneumology, University Medical Center, Mainz, Germany; 5 Anesthesiology, Pain and Palliative Medicine, Radboud University Medical Centre, Nijmegen, The Netherlands; 6 University Center for Palliative Care, Inselspital University Hospital Bern, University of Bern, Bern, Switzerland; 7 Institute of Social and Preventive Medicine, University of Bern, Bern, Switzerland; 8 Department of Metabolism, Digestion and Reproduction, Faculty of Medicine, Imperial College London, London, UK; 9 Department Family Medicine and Population Health (FamPop), Faculty of Medicine and Health Sciences, University of Antwerp, Antwerp, Belgium; 10 Department of Experimental, Diagnostic and Specialty Medicine, University of Bologna & Academy of the Sciences of Palliative Medicine, Bologna, Italy; 11 Department of Psychology, University of Bologna, Bologna, Italy

**Keywords:** Prognosis, primary health care, survival, palliative care

## Abstract

**Background:**

The Surprise Question (‘Would I be surprised if this patient died within 12 months?’) identifies patients in the last year of life. It is unclear if ‘surprised’ means the same for each clinician, and whether their responses are internally consistent.

**Aim:**

To determine the consistency with which the Surprise Question is used.

**Design:**

A cross-sectional online study of participants located in Belgium, Germany, Italy, The Netherlands, Switzerland and UK. Participants completed 20 hypothetical patient summaries (‘vignettes’). Primary outcome measure: continuous estimate of probability of death within 12 months (0% [certain survival]–100% [certain death]). A threshold (probability estimate above which Surprise Question responses were consistently ‘no’) and an inconsistency range (range of probability estimates where respondents vacillated between responses) were calculated. Univariable and multivariable linear regression explored differences in consistency. Trial registration: NCT03697213.

**Setting/participants:**

Registered General Practitioners (GPs). Of the 307 GPs who started the study, 250 completed 15 or more vignettes.

**Results:**

Participants had a consistency threshold of 49.8% (SD 22.7) and inconsistency range of 17% (SD 22.4). Italy had a significantly higher threshold than other countries (*p* = 0.002). There was also a difference in threshold levels depending on age of clinician, for every yearly increase, participants had a higher threshold. There was no difference in inconsistency between countries (*p* = 0.53).

**Conclusions:**

There is variation between clinicians regarding the use of the Surprise Question. Over half of GPs were not internally consistent in their responses to the Surprise Question. Future research with standardised terms and real patients is warranted.

**What is already known about this topic?**

- The Surprise Question (‘Would I be surprised if this patient died within 12 months?’) is a screening tool which is used to identify patients with palliative care needs.
- The Surprise Question alone is not a very accurate way to prognosticate.
- It is not known whether prognostication with the Surprise Question is difficult because clinicians are intrinsically poor prognosticators, because the Surprise Question is interpreted in different ways by different clinicians, of because clinicians themselves are inconsistent in their level of surprise.

**What this paper adds?**

- Our study suggests that the threshold probability, before a death causes surprise, varies across six European countries.
- Many GPs (including those with specialist palliative care training) are inconsistent about the probability of death that elicits surprise.

**Implications for practice, theory or policy**

- Further research is needed to understand how the Surprise Question is used in practice, and whether consistency and accuracy could be improved by modifying the Surprise Question, or by training GPs in its use.

## Background

The Surprise Question (‘Would I be surprised if this patient died within 12 months?’) is a screening question used to identify patients in their last year of life. It is recommended as part of several primary care prognostic tools such as the Necesidades Paliativas (NECPAL), Gold Standard Framework Proactive Indicator Guidance (GSF-PIG) and the Prospective Prognostic Planning tool.^1^

In the UK, the NHS Long term plan^2^ suggests a shift of care away from the acute setting, to the community. This will bring additional pressure to community services, where General Practitioners (GPs) are already responsible for identifying patients who would benefit from palliative care; either through adopting a palliative care approach, or by referral to specialist palliative care services. Across Europe, GPs are often the primary caregivers for frail elderly people with advanced illness and are often the gatekeepers of hospital care.

Using the Surprise Question may facilitate access to specialist palliative care services and funding, which help to improve the patient’s quality, and often quantity, of life.^3–7^ Current forecasts of population demographics over the next 50 years, both in the UK^8^ and across Europe,^9^ highlight that the population is ageing and living longer with more complex healthcare needs; adding to the complexity of GPs’ role in identifying patients who are in the last year of life. Timely identification within primary care, across Europe, of patients approaching the end of life has already been noted as a challenge,^10^ particularly in non-cancer groups^11^ and often GPs report waiting until the patient is very close to death before initiating conversations about end-of-life care.^12^ This delay can hamper the integration of patients’ preferences and needs in care planning, and in a ‘good death’ experience^13,14^ so it is critical to try to understand and improve how GPs complete this task.

The simplicity of the Surprise Question would seem to make it an attractive tool for screening patients for palliative care in comparison to other prognostic tools or needs assessments that take more time to complete and require more detailed information. The Surprise Question was not originally designed as a standalone prognostic tool but rather as an indicator of palliative care need.^15^ Nonetheless, the Surprise Question alone is frequently regarded as a simple and effective way of identifying patients who are in the last year of life and who thus might be expected to have higher palliative care needs.^16–18^ However, the accuracy of the S Surprise Question, when used as a method of predicting survival, is inconsistent.^16,19^ It is unclear if that is because clinicians are intrinsically poor prognosticators, or if there are specific problems with how the Surprise Question is interpreted by different clinicians. After all, a death that is ‘surprising’ to one clinician may not be ‘surprising’ to another.

The primary aim of this study was to examine how consistent General Practitioners are in their response to the Surprise Question by examining the ‘threshold’ level of surprise which triggers a consistent change from a positive (‘Yes, I would be surprised’) to a negative (‘No, I would not be surprised’) response. Secondary aims were to look at the range of inconsistency around this threshold (i.e. the range of probability values over which clinicians vacillate between ‘yes’ and ‘no’ responses to the Surprise Question), and to look at these differences across countries and by disease.

## Methods

This study follows the ‘STrengthening the Reporting of OBservational studies in Epidemiology’ (STROBE) reporting guidelines.^20^ A more detailed methodology is available from the study protocol.^21^ The study was registered on Clinicaltrials.gov (NCT03697213) prospectively on the 29/03/2019. This study gained ethics approval from University College London REC (09/08/2018, ref 8675/003), University of Antwerp REC (07/01/2019, ref 18/50/589), Rhineland-Palatinate’s General Medical Council (26/11/2018), Radboud University Medical Center REC (20/12/2018, ref 2018-4949), Bern Ethics Committee (29/08/2018, ref 2018-00710) and University of Bologna REC (25/01/2019, ref 12590).

### Study design

A cross-sectional online study.

### Setting

An online study of GPs’ predictions about survival outcomes for 20 hypothetical patient summaries.

### Participants

GPs from six countries were approached to participate in the study. A multi-national approach was chosen so that wider comparisons about the consistency of the Surprise Question could be drawn. The countries participating in this research all had familiarity with the use of the Surprise Question in primary care settings.

Eligible participants were:

1. Registered GPs in one of the participating countries (Flanders (Belgium), Germany, Italy, The Netherlands, Switzerland and United Kingdom).
2. Able to read and understand the language in which the study was presented to them.

If the inclusion criteria were not met, or participants declined to participate, they were excluded.

Recruitment methods varied in each country. In the UK, GPs were notified about the study in newsletters of Local Medical Committees, the Royal College of General Practitioners, or through word of mouth. In Italy, the GPs recruited were either part of an already established network of collaboration^22^ or by word of mouth. In the Netherlands, GPs were recruited via the network of GPs specialised in palliative care, the academic GP network of Radboudumc and word of mouth. In Flanders, GPs were recruited via the network of GPs specialised in palliative care, via the academic GP network of UAntwerp, via local GP peer review groups or word of mouth. In Germany, participants were recruited via the local and regional networks of physicians with an interest in palliative care, the local GP emergency service, the state academy for continuing medical education and training, or word of mouth. In Switzerland GP’s were recruited among participants of a basic training in palliative medicine before being exposed to the training. GP of the network of University Center for Palliative Care were recruited by mail, and word of mouth. The website was open to recruitment from 25/03/2019 to 01/03/2020.

### Development of the online environment

The online environment was developed by a database specialist (CT). It contained 20 patient summaries, or ‘vignettes’. The process used to construct the vignettes has previously been reported.^21^ The vignettes covered common diseases with which GPs would be familiar. The vignettes were constructed by the authorship group and were designed to represent a varied patient group, some of whom would typically be expected to die within 12 months, some expected to live longer, and some whose life expectancy was uncertain (see Supplemental Material 1 for more details about the structured content of the vignettes).

### Translation process

The study was translated from English to German, Italian and Flemish. The translation process adhered to European Organisation for Research and Treatment of Cancer (EORTC)^23^ guidelines.

### Procedure

On entering the online environment, eligible participants were asked to provide consent to participation; this included the option to receive feedback on their performance. If they consented, participants were asked to provide demographic information about themselves and their clinical experience.

**Figure 1.**
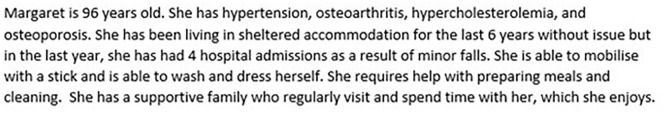
Example vignette.

Participants were asked to complete a practice vignette to familiarise themselves with the online environment and then 20 further vignettes (see Figure 1 for an example).

For each case, they were asked to provide a response for the following questions:

1. Would you be surprised if this patient were to die in the next 12 months? (Y/N) (The Surprise Question)
2. Would you be surprised if this patient were to remain alive after 12 months? (Y/N) (The second surprise question)
3. What do you think the probability is of this patient dying within the next 12 months? 0% (Certain survival)–100% (Certain death)
4. What do you think this patient needs? (select more than one if appropriate)

Question 4 was followed by a list of potential treatment or care options. Either the participant could select ‘Nothing’ or any of the options listed including ‘other’ (see Supplemental Material 2 for exact options). Question 2 and 4 were not the primary outcome of this research, and as such, the data will be reported elsewhere.

On completion of the vignettes, a debrief page thanked participants, reminded them of the study aims and offered them the option to withdraw their data. Participants were able to download a certificate of participation.

### Bias

To reduce the risk of attrition, participants were able to log out of the study as many times as needed, saving their progress, and returning at a more convenient time. To limit the impact of ordering effects, vignettes were presented in a randomised order to each participant.

### Study size

The sample size was based on estimating, to an acceptable level of precision, the probability of death which would trigger a consistent ‘lack of surprise’ in the participant (the ‘threshold’). As there was no existing evidence on this topic, we assumed that the probability of death which would trigger a change from surprise to lack of surprise would be 50%. Using this estimate, and aiming at a 4% margin of error (equivalently a precision of 8%), with a level of confidence of 95%, we aimed to recruit 600 participants in total (100 per country). Twenty vignettes were presented to each participant in order to keep the task burden to a minimum while collecting enough data to establish an individual’s threshold score.

### Statistical methods

Summary measures of participants were reported, including the number of missing observations for each characteristic. Participants who did not complete 15 or more vignettes were not included in the main analysis. The threshold level was calculated for each participant. This was defined as the probability at which responses to the Surprise Question consistently changed from ‘yes’ (I would be surprised if this patient died within the next year) to ‘no’ (I would not be surprised). To calculate the threshold, responses to the Surprise Question were examined across the 20 vignettes, ordered in accordance with increasing probability of dying attributed to them by participants.

The ‘range of inconsistency’ was defined as the difference between the lowest probability at which a participant responded with a ‘no’ to the Surprise Question and the threshold level (above which they consistently replied ‘yes’). During this range of probabilities, respondents’ answers vacillated between ‘yes’ and ‘no’. This is described in more detail in the Statistical Analysis Plan (NCT03697213) and study protocol.^21^ Participants were dichotomised into two groups according to their inconsistency range: those who were fully consistent (i.e. a single probability estimate that distinguished between ‘yes’ and ‘no’ responses); and those with at least some inconsistent responses (i.e. some switching between responses before settling down to a consistent answer).

Univariable linear regression analyses were performed to explore the differences, if any, in the threshold level and inconsistency range by country, participant demographics (age, gender) and clinical variables (extent of specialist palliative care post-graduate training, years of experience as a GP and frequency of use of the Surprise Question). Multivariable linear regression analyses were performed, to investigate the combination of variables on the threshold level and consistency range.

For each vignette, summary measures of the responses to the Surprise Question and the probability estimate were calculated using means (with standard deviations). The vignettes were ordered according to increasing frequency of adverse prognostic variables, grouped by disease category.

### Patient and public involvement

Members of the Marie Curie Expert VOICES group reviewed and informed the research proposal, design, and potential implications and meaning of the results in practice.

## Results

The study was started by 307 GPs, of whom 250 (81%) completed 15 or more vignettes and were included in the analyses. Excluded participants (*n* = 57) completed 4.3 (SD 2.5) vignettes. Of the 250 participants included in the analysis, the majority (*n* = 247, 99%) completed all 20 vignettes, one person (0.4%) completed 15 vignettes and two people (0.8%) completed 16 vignettes. Out of all of the responses, 113 (45.4%) were completely consistent, in that they changed from being surprised to unsurprised at a particular probability of dying and did not switch back to becoming surprised again as the estimated probability of death increased. Table 1 presents the summary measures of participants, including missing values, by country and by whether or not they were consistent in their responses.

**Table 1.** Participant characteristics.

*N*	Overall	Flanders	Germany	Italy	The Netherlands	Switzerland	UK	Inconsistent		Consistent	
	249^≠^	33	18	97	30	11	60	*n*	*n* (%)	*n*	*n* (%)
Gender								136		113	
Female	129 (51.8)	18 (54.6)	11 (61.1)	40 (41.2)	15 (50)	4 (36.4)	41 (68.3)		80 (58.8)		49 (43.4)
Male	120 (48.2)	15 (45.5)	7 (38.9)	57 (58.8)	15 (50)	7 (63.6)	19 (31.7)		56 (41.2)		67 (56.6)
Age (median, SD)	54 (41, 63)	52 (32, 60)	51.5 (49, 58)	62 (54, 66)	56.5 (47, 61)	44 (37, 63)	47 (38, 53)	135	56 (44, 63)	112	53 (40, 62)
Work setting*								134		112	
Acute hospital	5 (2)	1 (3.1)	1 (5.6)	0 (0)	1 (3.5)	2 (18.2)	0 (0)		2 (1.5)		3 (2.7)
Hospice	20 (8.1)	4 (12.5)	1 (5.6)	10 (10.3)	2 (6.9)	0 (0)	3 (5.1)		9 (6.7)		11 (9.8)
GP/community	226 (91.9)	28 (87.5)	15 (83.3)	96 (99)	25 (86.2)	5 (45.5)	57 (96.6)		112 (91)		104 (92.9)
Other	18 (7.3)	2 (6.3)	3 (16.7)	1 (1)	5 (17.2)	4 (36.4)	3 (5.1)		13 (9.7)		5 (4.5)
Postgraduate training^ǂ^								135		107	
Yes	78 (32.2)	11 (33.3)	14 (82.4)	19 (19.6)	18 (62.1)	5 (50)	11 (19.6)		47 (34.8)		31 (29)
No	164 (67.8)	22 (66.7)	3 (17.7)	78 (80.4)	11 (37.9)	5 (50)	45 (80.4)		88 (65.2)		76 (71)
Years/GP (median, IQR)	20.5 (9, 30.5)	13 (6, 32)	18.5 (8, 23)	25 (15, 38)	24.5 (17, 28)	5 (2, 30)	16 (8, 23)	136	22 (10.5, 32)	112	20 (8, 29)
Use of the Surprise Question											
Frequency of use of Surprise Question in clinical practice								136		112	
Daily	8 (3.2)	0 (0)	1 (5.6)	3 (3.1)	2 (6.7)	0 (0)	2 (3.4)		6 (4.4)		2 (1.8)
Weekly	27 (10.9)	5 (15.2)	1 (5.6)	5 (5.2)	3 (10)	1 (9.1)	12 (20.3)		13 (9.6)		14 (12.5)
Monthly	44 (17.7)	8 (24.2)	6 (33.3)	1 (1)	11 (36.7)	2 (18.2)	16 (27.1)		18 (13.2)		26 (23.2)
Less than monthly	92 (37.1)	15 (45.5)	3 (16.7)	50 (51.6)	6 (20)	3 (27.3)	15 (25.4)		48 (35.3)		44 (39.3)
Never	77 (31.1)	5 (15.2)	7 (38.9)	38 (39.2)	8 (26.7)	5 (45.5)	14 (23.7)		51 (37.5)		26 (23.2)
Confidence in prediction using Surprise Question								85		86	
Not at all	10 (5.9)	2 (7.1)	1 (9.1)	4 (6.8)	2 (9.1)	0 (0)	1 (2.2)		5 (5.9)		5 (5.8)
Not very	58 (33.9)	12 (42.9)	4 (36.4)	15 (25.4)	7 (31.8)	4 (66.7)	16 (35.6)		28 (32.9)		30 (34.9)
Fairly	89 (52.1)	13 (46.4)	6 (54.6)	29 (49.2)	13 (59.1)	2 (33.3)	26 (57.8)		48 (56.5)		41 (47.7)
Very	14 (8.2)	1 (3.6)	0 (0)	11 (18.6)	0 (0)	0 (0)	2 (4.4)		4 (4.7)		10 (11.6)

* Some clinicians worked in more than one setting, hence the % value.

≠ One person did not complete any demographic detail.

ǂ Postgraduate training in specialist palliative care.

### Threshold level and range of inconsistency

Participants had a threshold of 49.8% (SD 22.7); representing the probability above which respondents would consistently no longer be surprised if the patient described in the vignette were to have died within 1 year (Table 2). The threshold level varied by country, with the Netherlands having the lowest value of 40.6% (SD 26.0) and Italy having the highest at 57% (SD 21.9).

**Table 2.** Threshold values and ranges of inconsistency by country.

	*N*	Mean % (SD)	Median % (IQR)
Threshold			
Flanders	33	45.2 (24.5)	40 (30, 50)
Germany	18	50.3 (22.1)	50 (30, 60)
Italy	97	57.0 (21.9)	50 (40, 70)
The Netherlands	30	40.6 (26.0)	35 (20, 50)
Switzerland	11	48.2 (21.2)	50 (30, 50)
UK	60	45.2 (18.9)	40 (30, 50)
Total	250	49.8 (22.7)	50 (30, 65)
Range of inconsistency			
Flanders	33	13.3 (24.1)	0 (0, 15)
Germany	18	15.0 (24.9)	2.5 (0, 15)
Italy	97	20.4 (22.9)	10 (0, 30)
The Netherlands	30	16.5 (25.8)	2.5 (0, 20)
Switzerland	11	15.9 (19.6)	10 (0, 30)
UK	60	14.3 (18.6)	7.5 (0, 25)
Total	250	17.0 (22.4)	10 (0, 30)

The inconsistency range for participants overall was 17% (SD 22.4). Where participants gave an estimate between 33% and 50%, the response to the Surprise Question fluctuated; sometimes responding ‘yes’ and at other times ‘no’. The range of inconsistency varied across countries, with Flanders having the lowest level at 13% (SD 24.1) and Italy the highest at 20% (SD 22.9). Supplemental Material 3 gives a visual representation of the threshold and level of consistency for the group overall.

The univariable linear regression analysis indicated that participants in Italy had a significantly higher threshold than other countries (*p* = 0.002) (Table 3). There was also a difference in threshold levels depending on age of clinician, indicating that for every yearly increase in age, participants had a higher threshold. The multivariable regression shows that the relationship between threshold and country remained. Univariable linear regression analysis indicated there was no difference in the range of inconsistency between countries (*p* = 0.528). Participants who had received specialist palliative care postgraduate training had a slightly lower range of inconsistency than those who had no additional training; however, this difference was not significant. The multivariable analysis replicated these findings.

**Table 3.** Univariable and multivariable regression results exploring the threshold level.

	Threshold level		Inconsistency range	
	Univariable	Multivariable	Univariable	Multivariable
	Coeff. (95% CI)			
Country				
Flanders	Ref.	Ref.	Ref.	Ref.
Germany	5.1 (−7.6, 17.9)	3.8 (−10.6, 18.1)	1.7 (−11.3, 14.7)	−2 (−16.5, 12.4)
Italy	11.8 (3.1, 20.6)	11 (0.9, 21.2)	7.1 (−1.8, 16)	5.8 (−4.5, 16)
The Netherlands	−4.5 (−15.5, 6.5)	−5.5 (−17.4, 6.4)	3.1 (−8.1, 14.3)	1.4 (−10.6, 13.5)
Switzerland	3 (−12.1, 18.2)	4.5 (−12.2, 21.3)	2.6 (−12.9, 18)	2.7 (−14.1, 19.6)
United Kingdom	0 (−9.4, 9.5)	1.1 (−8.9, 11.2)	1 (−8.6, 10.6)	1.8 (−8.3, 12)
Specialist palliative care postgraduate training (ref = no)	1.6 (−4.6, 7.8)	4.8 (−2.1, 11.8)	4.4 (−1.8, 10.5)	6 (−1, 13)
Number years as GP	0.2 (−0.1, 0.4)	−0.1 (−0.6, 0.5)	0.1 (−0.1, 0.3)	−0.1 (−0.6, 0.5)
Age (years)	0.3 (0, 0.5)	0.2 (−0.4, 0.8)	0.2 (−0.1, 0.4)	0.2 (−0.4, 0.7)
Gender (ref = female)	−1 (−6.7, 4.7)	−3.6 (−9.9, 2.7)	−4.5 (−10.1, 1.1)	−6 (−12.4, 0.3)
Frequency of use				
Daily	2.4 (−14.2, 19.1)	1.2 (−15.8, 18.2)	7.7 (−8.8, 24.1)	4.6 (−12.5, 21.7)
Weekly	−3.8 (−13.8, 6.2)	1.3 (−9.7, 12.3)	−5.5 (−15.4, 4.4)	−2.2 (−13.3, 8.9)
Monthly	−5.6 (−14.1, 2.8)	−1.2 (−10.4, 7.9)	−6.7 (−15.1, 1.7)	−5.6 (−14.8, 3.6)
Less than monthly	2.7 (−4.2, 9.6)	3.3 (−3.8, 10.5)	−1.7 (−8.6, 5.1)	−0.2 (−7.5, 7)
Never	Ref.	Ref.	Ref.	Ref.^a^

a Adjusted for country, age, gender, specialist palliative care post-graduate training, years of experience as a GP and frequency of use of the Surprise Question.

### Analysis by vignettes

Table 4 presents the responses by vignette, grouped by health condition. Predicted probability of death within the next year ranged from 25% (SD 18.1) to 90% (SD 11.8). Overall, countries seemed to give similar probability estimates, with a mean difference of 14% between the estimates given. For example, each country considered that the patient in vignette 3 had an 88% or above chance of dying within the next 12 months. Patients with cancer or heart failure appeared to elicit the lowest levels of difference in probability estimates from GPs, and patients with chronic kidney disease (vignette 4) appeared to elicit the highest levels of disagreement.

**Table 4.** Responses to the Surprise Question and probability per vignette by country.

Disease	ID	Surprise Question		Probability of person dying within next 12 months.							Range*
		Yes	No	Overall	Flanders	Germany	Italy	The Netherlands	Switzerland	UK	
		*n* (%)		Mean (SD)							
Cancer	1	104 (41.9)	144 (58.1)	40.4 (20.7)	42.6 (21.4)	40.6 (20.7)	38.4 (21.4)	40 (21.7)	33.2 (11.2)	44.2 (19.8)	11.0
	2	8 (3.2)	241 (96.8)	70.6 (19)	65.5 (18.8)	72.6 (17.1)	70.6 (20.7)	64.9 (19.9)	71.8 (19)	75.4 (15)	10.5
	3	11 (4.4)	239 (95.6)	90.8 (11.8)	91.9 (10.5)	93.2 (6.9)	88.1 (14.6)	90.8 (12)	90.8 (8.2)	93.6 (7.6)	5.5
CKD	5	221 (88.4)	29 (11.6)	18 (21.3)	11.7 (9.9)	50.8 (31.2)	17.8 (21.3)	8.7 (7.8)	40.9 (29.2)	12.6 (11.8)	42.1
	7	235 (94.4)	14 (5.6)	18.8 (17.6)	12.5 (7.6)	19.3 (11.9)	24.1 (21.7)	13 (9.3)	21.8 (20.4)	15.6 (15.9)	11.6
	6	55 (22.1)	194 (77.9)	49.7 (21.6)	47.8 (22)	48.6 (18.1)	54.3 (20.5)	38.6 (22.5)	46.8 (17.1)	49.5 (22.6)	15.7
	4	31 (12.4)	219 (87.6)	61.5 (20.5)	58 (21.2)	68.6 (16.7)	62.1 (19.9)	55.7 (24.3)	57.3 (22)	63.8 (19.3)	12.9
Frailty	8	67 (26.9)	182 (73.1)	49.9 (21.5)	50.6 (25)	49.7 (21.7)	49.1 (20.6)	42.7 (19.5)	44.5 (18.1)	55.2 (22.1)	12.5
	10	55 (22.1)	194 (77.9)	48.9 (19.2)	48.6 (18.6)	54.4 (16.4)	49 (19.8)	42.3 (18.3)	51.8 (13.5)	50 (20.3)	12.1
	9	6 (2.4)	243 (97.6)	80.5 (17)	78.5 (19.8)	79.1 (17.3)	78 (17.7)	79.3 (15.8)	79.1 (20.7)	87 (12.2)	9.0
Heart	13	81 (32.4)	169 (67.6)	45.3 (20.2)	43.5 (20.7)	44.7 (19.2)	47.1 (22.5)	38.3 (17.5)	39.1 (18.1)	48.2 (17.5)	9.9
	12	41 (16.4)	209 (83.6)	53.4 (21.6)	45 (22.4)	51.7 (16.8)	58.8 (22.6)	46.5 (21.5)	50.5 (16.8)	53.9 (19.8)	13.8
	11	85 (34)	165 (66)	43.7 (20.9)	41.2 (22.6)	44.4 (17.3)	41.7 (20.5)	41.8 (19.8)	50.5 (11.9)	47.7 (23.2)	9.3
Dementia	15	28 (11.3)	220 (88.7)	63.2 (22.4)	57.1 (21)	67.5 (13.9)	58.4 (24.4)	69.5 (19.8)	69.5 (15.7)	68.3 (22.3)	12.4
	14	63 (25.2)	187 (74.8)	51.4 (22)	45.8 (22.9)	50.6 (16.8)	52.8 (22.7)	39.6 (20.7)	48.6 (17.3)	58.8 (20.4)	19.2
	16	9 (3.6)	240 (96.4)	69.6 (19.3)	64.5 (19.7)	67.4 (16)	69.3 (20.1)	68.4 (16.8)	77.2 (13.3)	72.6 (20.4)	12.7
COPD	18	199 (79.9)	50 (20.1)	25 (18.2)	24.9 (19.4)	21.9 (11.1)	28.8 (20)	19.3 (17.2)	30.9 (17.4)	21.6 (15.4)	11.6
	20	116 (46.4)	134 (53.6)	39.9 (21.5)	36.5 (21.8)	42.8 (19)	38.2 (22.7)	32.4 (19)	50 (22.7)	45.5 (19.8)	17.6
	19	115 (46)	135 (54)	37.7 (21.4)	31.2 (19)	36.4 (18.2)	34.6 (21.2)	35.7 (22.6)	47.7 (18.1)	45.6 (21.8)	16.5
	17	83 (33.3)	166 (66.7)	42.3 (20.5)	35.8 (17.5)	45 (21.5)	46.1 (20.3)	38.7 (21.5)	43.2 (18.3)	40.6 (21.1)	10.3

* Range: the difference between the highest and lowest probability estimates across the countries.

## Discussion

### Main findings

There was a significant difference in how GPs in different countries responded to the Surprise Question. The overall threshold level of surprise was an estimated probability of death within the next year of 49.8%, at which point ‘Yes, I would be surprised’ typically became ‘No, I would not be surprised’. This suggests that GPs interpret a negative response to the Surprise Question as being equivalent to a less than or equal to 50/50 chance of that patient surviving for 1 year. GPs in Italy had a higher threshold for the Surprise Question, implying that they might not consider palliative care for patients until the probability of death within the next year was greater than 50/50; whereas GPs in the Netherlands were likely to be ‘unsurprised’ even when the probability of death was estimated at 40%, suggesting that they might consider more patients as being in the last year of life and in need of palliative care.

### What this study adds

Timely identification of patients who would benefit from palliative care by GPs is essential, as it allows anticipatory care planning to relieve symptoms and to prevent future symptoms and problems.^24^ A meta-analysis of 12 trials has shown that palliative care improved the quality of life of patients, and the effect seemed to be marginally larger for patients with cancer and those who received specialist palliative care early.^25^ This research highlights that GPs give more similar survival estimates for patients with cancer or heart disease than for other conditions. Previous research has shown that GPs are more likely to include patients with cancer in palliative care registers compared to non-cancer patients.^26^ A potential explanation for this disparity could be that GPs are less familiar with caring for patients, such as those on dialysis, until nearer the ends of their lives.

This research identified differences between countries in their use of the Surprise Question. Differences and inconsistencies in the provision of palliative care have been observed amongst European countries.^27,28^ A European monitoring study showed that GPs had a proactive role in the delivery of primary palliative care in the Netherlands, whereas discussions regarding incurability of illness and life expectation took place significantly less often in Spain and Italy compared to Flanders and the Netherlands.^27^ The identification of, and response to, training needs of GPs and the palliative care needs of patients needs to be adapted to their respective cultural, social, healthcare and spiritual contexts.

Not only was there variability between different GPs in different countries about how surprised they would be about whether patients will die within the next year, but GPs’ responses were often internally inconsistent; sometimes expressing surprise and other times a lack of surprise, irrespective of the relative likelihoods that they attributed to patients dying. This lack of consistency suggests that GPs maybe interpreting the Surprise Question in different ways from each other, and sometimes individual GPs may be interpreting it in different ways for different patients on their own caseload. GPs answers to the Surprise Question are likely to be determined by more than just their prognostic estimates and may be influenced by their own willingness to refer to specialist palliative care services, their own perception regarding what role palliative care services have, or to recognise unmet palliative care needs.

### Strengths and limitations

This study found that 45% (113/250) of GPs were completely consistent with their responses to the Surprise Question: their predictions about the probability of death increased, and then at a particular point they consistently switched from being surprised to being not surprised. Overall, however, participants had a range of inconsistency of 17% (SD 22.4): this represented the range of probabilities over which their responses switched between ‘yes’ and ‘no’.

Since the Surprise Question is based on the subjective responses of individual clinicians, it is pertinent to know how consistent GPs are in their use of this tool. This is the first study to look at the consistency of use of the Surprise Question by GPs across multiple countries. However, the comparative analyses between countries need to be treated with caution. The study did not reach the originally planned sample size, and thus the confidence limits around our estimates of threshold values and inconsistency ranges were larger than we had anticipated. The study was conducted without dedicated funding and relied on academic collaboration. There were differences in the recruitment strategies in different countries and the national samples may not be representative or directly comparable. There is also the possibility that some of the differences may have arisen as a result of language differences or cultural factors, rather than because of specific differences in the use or interpretation of the Surprise Question. The sampling technique was not random, however the aim of the study was to investigate internal consistency

The vignettes did not describe real patients and so it is not possible to calculate how accurate the GPs predictions were. However, the purpose of the research was not to evaluate the accuracy of the Surprise Question, but rather to evaluate how it is interpreted by different clinicians and the consistency with which it is used. Nonetheless, the degree of agreement in responses between professionals and the alignment between estimates, disease categories and severity of health conditions, provides some evidence in support of the clinical realism of the vignettes.

## Conclusion

The Surprise Question was previously known to have variable accuracy.^16,19^ The current study has also shown that 55% of clinicians’ responses are not internally consistent. It is possible that the accuracy and the consistency of the Surprise Question as a prognostic tool could be improved with greater standardisation of terms (e.g. defining ‘surprise’, as when a death occurs and the expected probability is less than 50%). However, our study suggests that in its current format the Surprise Question is not suitable for use for prognostication. Future research, using real cases and after agreeing standardisation of terms, would help to evaluate the relationship between prognostic accuracy and consistency and to investigate what could be done to improve them.

## Supplemental Material

sj-docx-1-pmj-10.1177_02692163211048340 – Supplemental material for An online international comparison of palliative care identification in primary care using the Surprise Question Supplemental material, sj-docx-1-pmj-10.1177_02692163211048340 for An online international comparison of palliative care identification in primary care using the Surprise Question by Nicola White, Linda JM Oostendorp, Victoria Vickerstaff, Christina Gerlach, Yvonne Engels, Maud Maessen, Christopher Tomlinson, Johan Wens, Bert Leysen, Guido Biasco, Sofia Zambrano, Steffen Eychmüller, Christina Avgerinou, Rabih Chattat, Giovanni Ottoboni, Carel Veldhoven and Patrick Stone in Palliative Medicine

sj-jpg-1-pmj-10.1177_02692163211048340 – Supplemental material for An online international comparison of palliative care identification in primary care using the Surprise Question Supplemental material, sj-jpg-1-pmj-10.1177_02692163211048340 for An online international comparison of palliative care identification in primary care using the Surprise Question by Nicola White, Linda JM Oostendorp, Victoria Vickerstaff, Christina Gerlach, Yvonne Engels, Maud Maessen, Christopher Tomlinson, Johan Wens, Bert Leysen, Guido Biasco, Sofia Zambrano, Steffen Eychmüller, Christina Avgerinou, Rabih Chattat, Giovanni Ottoboni, Carel Veldhoven and Patrick Stone in Palliative Medicine

sj-jpg-2-pmj-10.1177_02692163211048340 – Supplemental material for An online international comparison of palliative care identification in primary care using the Surprise Question Supplemental material, sj-jpg-2-pmj-10.1177_02692163211048340 for An online international comparison of palliative care identification in primary care using the Surprise Question by Nicola White, Linda JM Oostendorp, Victoria Vickerstaff, Christina Gerlach, Yvonne Engels, Maud Maessen, Christopher Tomlinson, Johan Wens, Bert Leysen, Guido Biasco, Sofia Zambrano, Steffen Eychmüller, Christina Avgerinou, Rabih Chattat, Giovanni Ottoboni, Carel Veldhoven and Patrick Stone in Palliative Medicine

sj-pdf-1-pmj-10.1177_02692163211048340 – Supplemental material for An online international comparison of palliative care identification in primary care using the Surprise Question Supplemental material, sj-pdf-1-pmj-10.1177_02692163211048340 for An online international comparison of palliative care identification in primary care using the Surprise Question by Nicola White, Linda JM Oostendorp, Victoria Vickerstaff, Christina Gerlach, Yvonne Engels, Maud Maessen, Christopher Tomlinson, Johan Wens, Bert Leysen, Guido Biasco, Sofia Zambrano, Steffen Eychmüller, Christina Avgerinou, Rabih Chattat, Giovanni Ottoboni, Carel Veldhoven and Patrick Stone in Palliative Medicine
