# Supplementary material for: An online international comparison of palliative care identification in primary care using the Surprise Question
Source: Palliat Med. 2021 Oct 1;36(1):142–51. doi: 10.1177/02692163211048340 (PMC8796152; doi:10.1177/02692163211048340)
Supplement: sj-docx-1-pmj-10.1177_02692163211048340 – Supplemental material for An online international comparison of palliative care identification in primary care using the Surprise Question [file sj-docx-1-pmj-10.1177_02692163211048340.docx]

Supplementary File 1 Structured content for the vignettes

| **Vignette** | **Diagnosis** | **Gender** | **Age group** | **Disease severity** | **Co-morbidities** | **# unplanned hospital adms/year** | **AKPS** | **weight loss/6 months** | **Sentinel Event*** |
| --- | --- | --- | --- | --- | --- | --- | --- | --- | --- |
| Practice | Frailty | Female | 80-100 | moderate | multiple | 2+ | 40-60 | <10% | No |
| 1 | Cancer | Male | 60-79 | mild | none or 1 | 0-1 | 70-100 | >10% | Yes |
| 2 | Cancer | Male | 80-100 | moderate | multiple | 2+ | 40-60 | <10% | No |
| 3 | Cancer | Female | 80-100 | severe | multiple | 2+ | 10 to 30 | <10% | No |
| 4 | CKD | Male | 40-60 | severe | multiple | 0-1 | 40-60 | >10% | Yes |
| 5 | CKD | Male | 60-79 | mild | none or 1 | 2+ | 40-60 | <10% | Yes |
| 6 | CKD | Female | 40-60 | moderate | multiple | 0-1 | 10 to 30 | >10% | No |
| 7 | CKD | Female | 60-79 | moderate | none or 1 | 2+ | 70-100 | <10% | Yes |
| 8 | Frailty | Male | 60-79 | moderate | none or 1 | 0-1 | 40-60 | >10% | Yes |
| 9 | Frailty | Male | 80-100 | severe | multiple | 2+ | 10 to 30 | <10% | Yes |
| 10 | Frailty | Female | 80-100 | moderate | multiple | 2+ | 40-60 | <10% | No |
| 11 | Heart | Male | 60-79 | moderate | multiple | 0-1 | 10 to 30 | >10% | No |
| 12 | Heart | Male | 80-100 | moderate | none or 1 | 2+ | 70-100 | <10% | Yes |
| 13 | Heart | Female | 80-100 | mild | none or 1 | 2+ | 40-60 | <10% | No |
| 14 | Dementia | Male | 80-100 | moderate | multiple | 2+ | 40-60 | <10% | No |
| 15 | Dementia | Female | 60-79 | moderate | none or 1 | 0-1 | 40-60 | >10% | Yes |
| 16 | Dementia | Female | 80-100 | severe | multiple | 2+ | 10 to 30 | <10% | No |
| 17 | COPD | Female | 40-60 | severe | multiple | 0-1 | 40-60 | >10% | No |
| 18 | COPD | Male | 60-79 | mild | none or 1 | 2+ | 40-60 | <10% | Yes |
| 19 | COPD | Male | 40-60 | moderate | multiple | 0-1 | 10 to 30 | >10% | No |
| 20 | COPD | Female | 60-79 | moderate | none or 1 | 2+ | 70-100 | <10% | Yes |

*Sentinel event: bereavement or change of living environment e.g. relocated to nursing home; CKD: Chronic Kidney Disease; COPD: Chronic obstructive pulmonary disease; AKPS: Australia-modified Karnofsky Performance Status.
