# Supplementary figures and images for: An online international comparison of palliative care identification in primary care using the Surprise Question
Source: Palliat Med. 2021 Oct 1;36(1):142–51. doi: 10.1177/02692163211048340 (PMC8796152; doi:10.1177/02692163211048340)

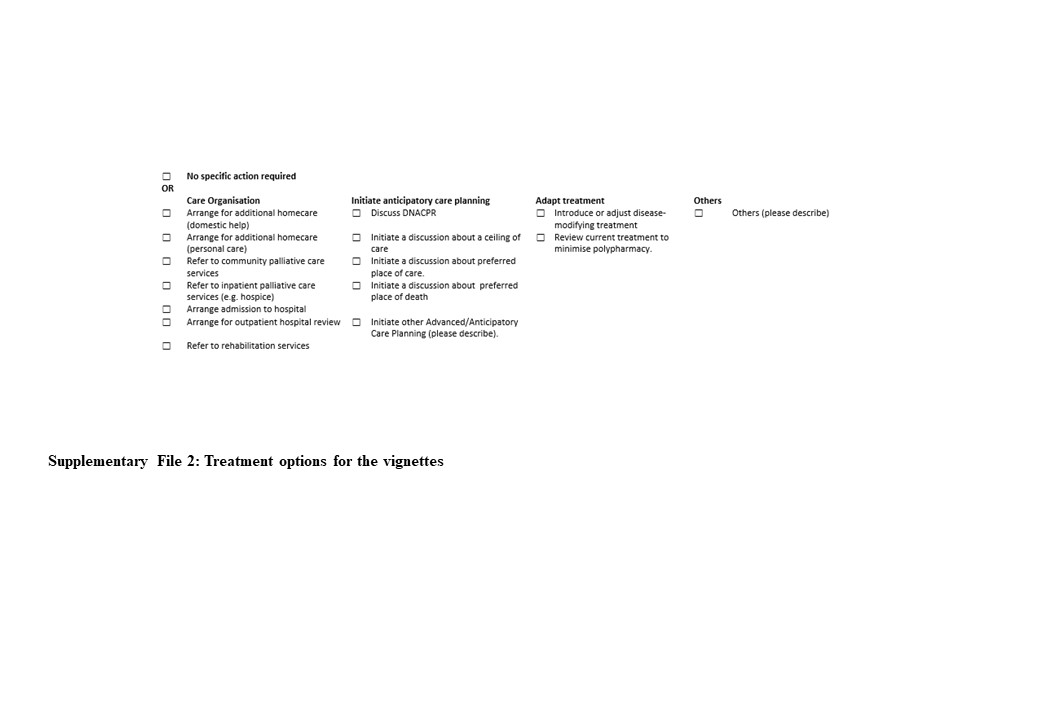

Supplement: sj-jpg-1-pmj-10.1177_02692163211048340 – Supplemental material for An online international comparison of palliative care identification in primary care using the Surprise Question [file sj-jpg-1-pmj-10.1177_02692163211048340.jpg]

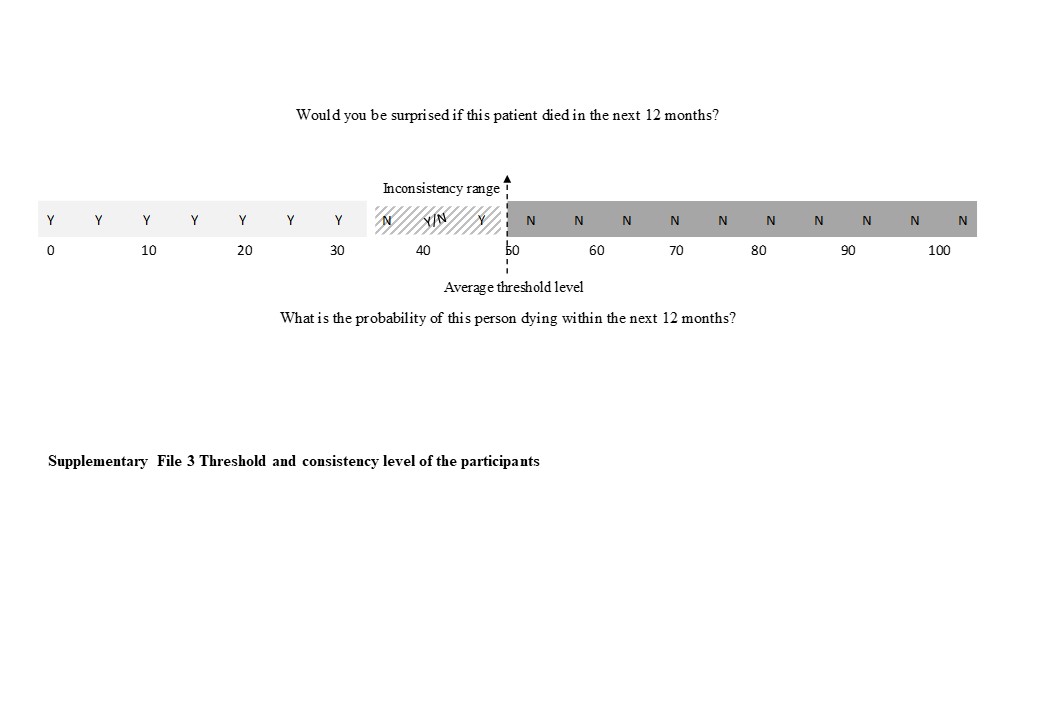

Supplement: sj-jpg-2-pmj-10.1177_02692163211048340 – Supplemental material for An online international comparison of palliative care identification in primary care using the Surprise Question [file sj-jpg-2-pmj-10.1177_02692163211048340.jpg]
