# Supplementary material for: An online international comparison of palliative care identification in primary care using the Surprise Question
Source: Palliat Med. 2021 Oct 1;36(1):142–51. doi: 10.1177/02692163211048340 (PMC8796152; doi:10.1177/02692163211048340)
Supplement: sj-pdf-1-pmj-10.1177_02692163211048340 – Supplemental material for An online international comparison of palliative care identification in primary care using the Surprise Question [file sj-pdf-1-pmj-10.1177_02692163211048340.pdf]

Supplementary File 1 Structured content for the vignettes

Vignette	Diagnosis	Gender	Age group	Disease severity	Co-morbidities	# unplanned hospital adms/year	AKPS	weight loss/6 months	Sentinel Event*
Practice	Frailty	Female	80-100	moderate	multiple	2+	40-60	<10%	No
1	Cancer	Male	60-79	mild	none or 1	0-1	70-100	>10%	Yes
2	Cancer	Male	80-100	moderate	multiple	2+	40-60	<10%	No
3	Cancer	Female	80-100	severe	multiple	2+	10 to 30	<10%	No
4	CKD	Male	40-60	severe	multiple	0-1	40-60	>10%	Yes
5	CKD	Male	60-79	mild	none or 1	2+	40-60	<10%	Yes
6	CKD	Female	40-60	moderate	multiple	0-1	10 to 30	>10%	No
7	CKD	Female	60-79	moderate	none or 1	2+	70-100	<10%	Yes
8	Frailty	Male	60-79	moderate	none or 1	0-1	40-60	>10%	Yes
9	Frailty	Male	80-100	severe	multiple	2+	10 to 30	<10%	Yes
10	Frailty	Female	80-100	moderate	multiple	2+	40-60	<10%	No
11	Heart	Male	60-79	moderate	multiple	0-1	10 to 30	>10%	No
12	Heart	Male	80-100	moderate	none or 1	2+	70-100	<10%	Yes
13	Heart	Female	80-100	mild	none or 1	2+	40-60	<10%	No
14	Dementia	Male	80-100	moderate	multiple	2+	40-60	<10%	No
15	Dementia	Female	60-79	moderate	none or 1	0-1	40-60	>10%	Yes
16	Dementia	Female	80-100	severe	multiple	2+	10 to 30	<10%	No
17	COPD	Female	40-60	severe	multiple	0-1	40-60	>10%	No
18	COPD	Male	60-79	mild	none or 1	2+	40-60	<10%	Yes
19	COPD	Male	40-60	moderate	multiple	0-1	10 to 30	>10%	No
20	COPD	Female	60-79	moderate	none or 1	2+	70-100	<10%	Yes

*Sentinel event: bereavement or change of living environment e.g. relocated to nursing home; CKD: Chronic Kidney Disease; COPD: Chronic obstructive pulmonary disease; AKPS: Australia-modified Karnofsky Performance Status.
